# Use of centrifugal systems for investigating water flow processes in unsaturated soils

**DOI:** 10.1038/s41598-022-18103-0

**Published:** 2022-08-12

**Authors:** Huanhuan Qin

**Affiliations:** 1 State Key Laboratory of Nuclear Resources and Environment, East China University of Technology, Nanchang, 330013 Jiangxi China; 2 School of Water Resources and Environmental Engineering, East China University of Technology, Nanchang, 330013 Jiangxi China

**Keywords:** Hydrology, Environmental impact

## Abstract

Centrifugal modelling, both physical and numerical, has been used for studying groundwater flow and transport processes in the past. However, there was disagreement in previous studies whether numerical models can be used in simulating centrifugal systems under unsaturated flow condition. In the present study, a numerical model based on Richards’ equation was developed to predict one-dimensional unsaturated flow in centrifugal systems. The validity of the model was tested using data from physical models in four published benchmark problems. The ability of the numerical model to close mass balance was also tested. It was shown that the newly developed numerical model was able to recreate the four benchmark problems quite successfully, indicating that using such a model under unsaturated flow condition is feasible. The mass conservation result shows that the model is more sensitive to spatial grid resolution than to specified temporal step. Therefore, fine spatial discretization is suggested to ensure the simulation quality. Additionally, adaptive temporal time stepping method can be used to improve the computational efficiency. It was found that the dimensionless factors used for scaling physical dimensions by 1/N, seepage velocity by N, and temporal dimension by 1/N^2^ were useful parameters for scaling centrifugal systems.

## Introduction

Natural water flow in the upper layers of the subsurface, namely the unsaturated zone (or the vadose zone), contains liquid, solid and gaseous phases at the same time, which makes it challenging to compute water flow and solute transport processes in such systems. Simulating the unsaturated flow processes in a controlled laboratory environment using both physical and numerical models is essential to understand the key processes and subsequently develop strategies for managing the soil–water systems^1^. Centrifugal systems are physical-scale models typically used in studies of flow processes in porous media and they offer several advantages over the other types of physical scale models^2–4^. First, water flows are typically slow in unsaturated porous media flow systems, but a centrifuge can accelerate the speed of flow to ensure rapid data collection; second, the centrifugal system can be used to predict the migration behavior of full scale prototypes through the similarity of related parameters; third, centrifugal systems allow to adjust a number of flow parameters that can be scaled to predict the field scale behavior, and finally, these models are suitable for testing both 2- and 3-dimensional flow schemes with appropriate boundary conditions. In recent years, centrifugal systems have been successfully used in studies of unsaturated flow processes^5–8^ and solute transport of various materials in unsaturated soil^9–13^. Other types of applications of the centrifugal systems have been used for simulating transient water flow in earth embankments^3^ and for determining the characteristic parameters of saturated/unsaturated soil systems^14,15^.

A centrifugal system can simulate the natural groundwater flow processes with the help of a centrifuge. The key step in employing such system is to develop a link between the full-scaled model (the "prototype") and the centrifugal system (downscaled from the prototype)^16^. One of the most critical parameters when establishing such a link is the scale factor, *N*, which represents the number of times the gravity (*g*) is used while centrifuging the samples. If a centrifugal system with size 1*/N* of a prototype operates with *N* times of gravity, the two effects will cancel each other and the generated stress by self-weight in the centrifuge will be the same as in the prototype. Studies have investigated the use of the scale factor for unsaturated flow in soil using dimensionless analysis^2,3,17^ and inspectional analysis^17,18^. In addition, the scale factor can also be obtained by fitting parameters of a numerical model to a physical centrifugal system^16^.

The currently available approaches for obtaining the scale factor have several limitations. The dimensionless analysis and the inspectional analysis assume the centrifugal acceleration to be uniformly distributed throughout the centrifugal system. The parameter fitting approach proposed by Dell'avanzi et al.^16^ can only be applied for one-dimensional steady-state problems. Furthermore, some studies suggest that centrifugal models may only be applied in saturated systems, since it is very difficult to obtain the scale factor for unsaturated conditions when the suction gradient is dominant^19,20^. Therefore, it is necessary to further explore the validity of using centrifugal systems to study unsaturated flow systems.

The objective of this study is to develop a modeling approach to test the hypothesis that centrifugal systems can be used for investigating unsaturated flow processes. The study also further tests the validity of using different types of scale factors while studying unsaturated flow processes using centrifugal systems.

## 1D centrifugal model

### Governing equation

In a centrifuge, unsaturated flow occurs under gravity forces and energy gradients are induced by the centrifugal forces. Several studies have proposed mathematical theories for modeling this system^21,22^. In the following sections, we review the assumptions involved in developing these theories and present a systematic procedure for deriving the governing equations.

#### Definition of water potential

**Figure 1:**
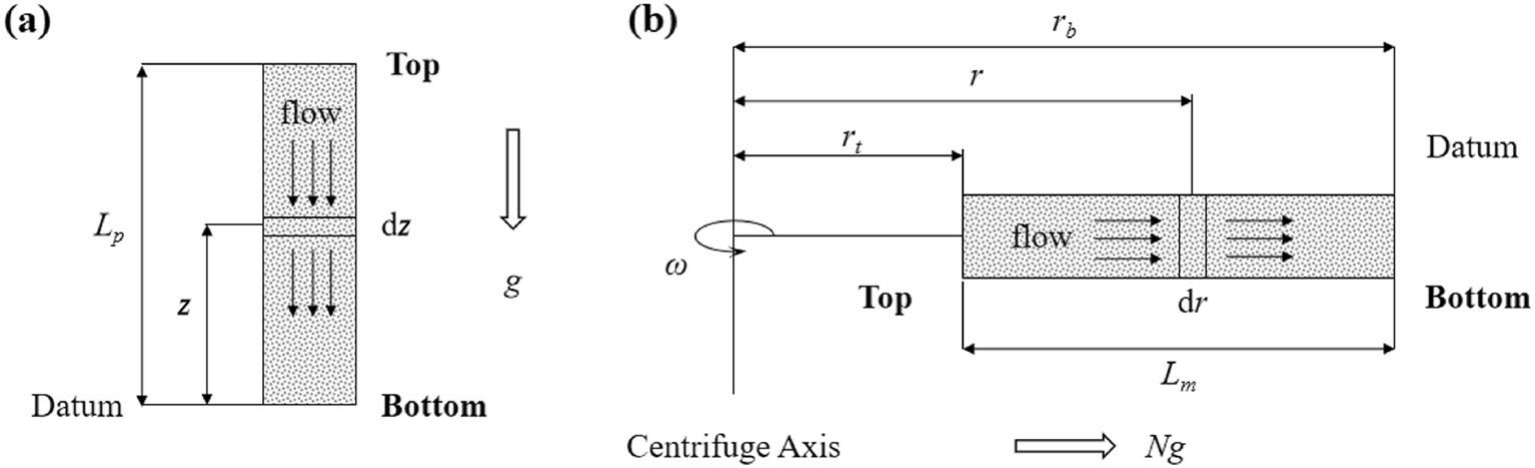
Schematic illustrations of one-dimensional flow taking place in (**a**) natural condition (i.e., prototype) and (**b**) centrifugal model. *L*_*p*_ is the length of the prototype, *z* is the height of current position from column bottom, d*z* is the infinitesimal increment in z direction, *g* is the gravitational acceleration, while *r*_*t*_ is the model top radius, *r*_*b*_ is the model bottom radius, *r* is the radius of current position, d*r* is the infinitesimal increment of *r*, and *L*_*m*_ is the length of the centrifugal column.

Figure 1 shows a schematic diagram of one-dimensional flow taking place in a centrifugal system. Flow is driven by a gradient in water potential, which is a function of elevation potential, kinetic energy and matric potential. The net water potential per unit mass at a point within a centrifugal system can be expressed as^23^:

1 $$\Phi = P_{e} + \frac{{\left( {v_{m} /\theta_{m} } \right)^{2} }}{2} + \frac{{\psi_{m} }}{\rho }$$

where Φ is water potential per unit mass [L^2^ T^−2^], *P*_*e*_ is centrifugal elevation potential per unit mass[L^2^ T^−2^], *v* is Darcy velocity [L T^−1^], *θ* is water content [L^3^ L^−3^], *ψ* is matric suction [M L^−1^ T^−2^], *ρ* is water density [M L^−3^], and subscript *m* denotes “centrifugal model” (hereafter the same). The seepage velocity (i.e. the ratio between Darcy velocity and water content) in the centrifugal acceleration field is generally small^24,25^, leading to kinetic energy negligible. In this case, the water potential becomes:

2 $$\Phi = P_{e} + \frac{{\psi_{m} }}{\rho }$$

The centrifugal acceleration is a function of radial distance and angular speed, and it is calculated in the following equation^19,26^:

3 $$a = r\omega^{2}$$

where *a* is centrifugal acceleration [L T^−2^], *r* is radius [L], *ω* is angular speed [T^−1^]. Equation 3 shows that centrifugal acceleration is distributed along the vertical direction of the centrifugal system. This distribution is noticeably different from the distribution of natural gravitational acceleration (almost uniform near the earth surface), so the elevation potential of water in the system must be derived from the most basic definition of elevation potential. Centrifugal elevation potential is equal to the work done to overcome centrifugal force, and elevates one unit mass of water from the datum to the current position. The mathematical expression for centrifugal elevation potential at *r* from the system bottom (set as the datum) is:

4 $$P_{e} = - \int_{{r_{b} }}^{{r^{\prime}}} {a{\kern 1pt} } {\text{d}}r$$

where *r*_*b*_ is the distance between the system bottom and the centrifuge axis [L]. Substitute Eq. (3) into Eq. (4), and do a definite integration:

5 $$P_{e} = \frac{1}{2}\omega^{2} \left( {r_{b}^{2} - r^{2} } \right)$$

Combining Eqs. (2) and (5), the water potential for unsaturated flow taking place in a centrifugal system can be wrote in the form of:

6 $$\Phi = - \frac{1}{2}\omega^{2} \left( {r^{2} - r_{b}^{2} } \right) + \frac{{\psi_{m} }}{\rho }$$

This expression of water potential is similar to the one used by Nimmo et al.^22^ and Conca and Wright^27^. The difference is the choice of datum, which was set to be the centrifuge axis in those equations.

#### Darcy’s law

Darcy’s law describes the internal relationship between flow and gradient of water potential. Darcy’s law for flow in a centrifugal system has been verified by many studies^8,24,25,28^, and it can be shown in the form of

7 $$v_{m} = \frac{{K(\psi )_{m} }}{g}\frac{\partial \Phi }{{\partial r}}$$

where *v* is seepage velocity [L T^−1^], *K*(*ψ*) is unsaturated hydraulic conductivity [L T^−1^], *g* is gravitational acceleration [L T^−2^]. Substitute Eq. (6) into Eq. (7) and rearrange it, the Darcy’s law for unsaturated flow under centrifugal acceleration can be deduced as:

8 $$v_{m} = - \frac{{K(\psi )_{m} }}{\rho g}\left[ {\rho \omega^{2} r - \frac{{\partial \psi_{m} }}{\partial r}} \right]$$

#### Richards’ equation for centrifugal modeling

Considering a control volume of a centrifugal system (shown in Fig. 1b), the principle of continuity leads to^16^:

9 $$\frac{{\partial \theta_{m} }}{{\partial t_{m} }} = \frac{{\partial v_{m} }}{\partial r}$$

where *t* represents centrifugal modeling time [T], *θ* is water content [L^3^ L^−3^]. Substituting Eq. (8) into Eq. (9) results in:

10 $$\frac{{\partial \theta_{m} }}{{\partial t_{m} }} = \frac{\partial }{\partial r}\left[ { - \frac{{K(\psi )_{m} }}{\rho g}\left( {\rho \omega^{2} r - \frac{{\partial \psi_{m} }}{\partial r}} \right)} \right]$$

Equation 10 is the general governing equation of unsaturated flow^15^. It describes the one-dimensional flow through unsaturated soils under a centrifugal acceleration system. By rearranging Eq. (10), unsaturated flow in the centrifugal system can be expressed as:

11 $$\frac{{\partial \theta_{m} }}{{\partial t_{m} }} = \frac{1}{\rho g}\frac{\partial }{\partial r}\left[ {K(\psi )_{m} \frac{{\partial \psi_{m} }}{\partial r}} \right] - \frac{{\omega^{2} }}{g}\frac{\partial }{\partial r}\left[ {K(\psi )_{m} r} \right]$$

Equations (10) and (11) are Richards’ equation in two different forms. A detailed numerical method for solving the centrifuge equation in the form of Eq. (11) is provided.

### Richards’ equation of the prototype

Unsaturated flow taking place under normal gravitational acceleration is taken into account in the prototype (see Fig. 1a). In order to set an inspection standard for centrifugal modeling, a standard Richards’ equation of the prototype in the following form was solved:

12 $$\frac{{\partial \theta_{p} }}{{\partial t_{p} }} = \frac{{\partial K(\psi )_{p} }}{\partial z} + \frac{1}{\rho g}\frac{\partial }{\partial z}\left[ {K(\psi )_{p} \frac{{\partial \psi_{p} }}{\partial z}} \right]$$

where *z* is the height from the column bottom [L]. The other symbols in Eq. (12) are the same as those presented in the derivation process of Richards’ equation of centrifugal modeling (RECM), and the subscript *p* denotes “prototype” (hereafter the same). Equation (12) is a mixed form of the Richards’ equation^29^ and its simulation results are set as the inspection standard to verify centrifugal modeling.

### g-Level

The ratio between effective acceleration and gravity is termed as *g*-level, which is always noted as *N*. Because centrifugal acceleration is a function of radius and angular speed, an error caused by stress distribution exists between the centrifugal model and the prototype. Taylor pointed out that the error is minimal if the stress at the 2/3 height of the model is as same as the corresponding point of the prototype^30^, so *N* can be obtained using the following equation:

13 $$N = \frac{{\omega^{2} }}{g}\left( {r_{b} - \frac{2}{3}L_{m} } \right)$$

where *L*_*m*_ is the length of the centrifugal system [L].

### Numerical solution

Unsaturated flow can be predicted by solving the RECM numerically, and several numerical methods have already been published in literatures^7,15^ in the past three decades. Nimmo^7^ developed a direct solver of the RECM, and the numerical simulations using independent soil hydraulic properties successfully matched the measured results of transient flow experiments. Šimůnek and Nimmo^15^ modified the Hydrus software package to directly or inversely simulate unsaturated flow in a transient centrifugal field. In this study, we reviewed and adapted some of these numerical schemes and presented a detailed step-by-step approach that can be directly used by others. Furthermore, we assembled a set of benchmarks to rigorously validate the numerical solution.

**Figure 2:**
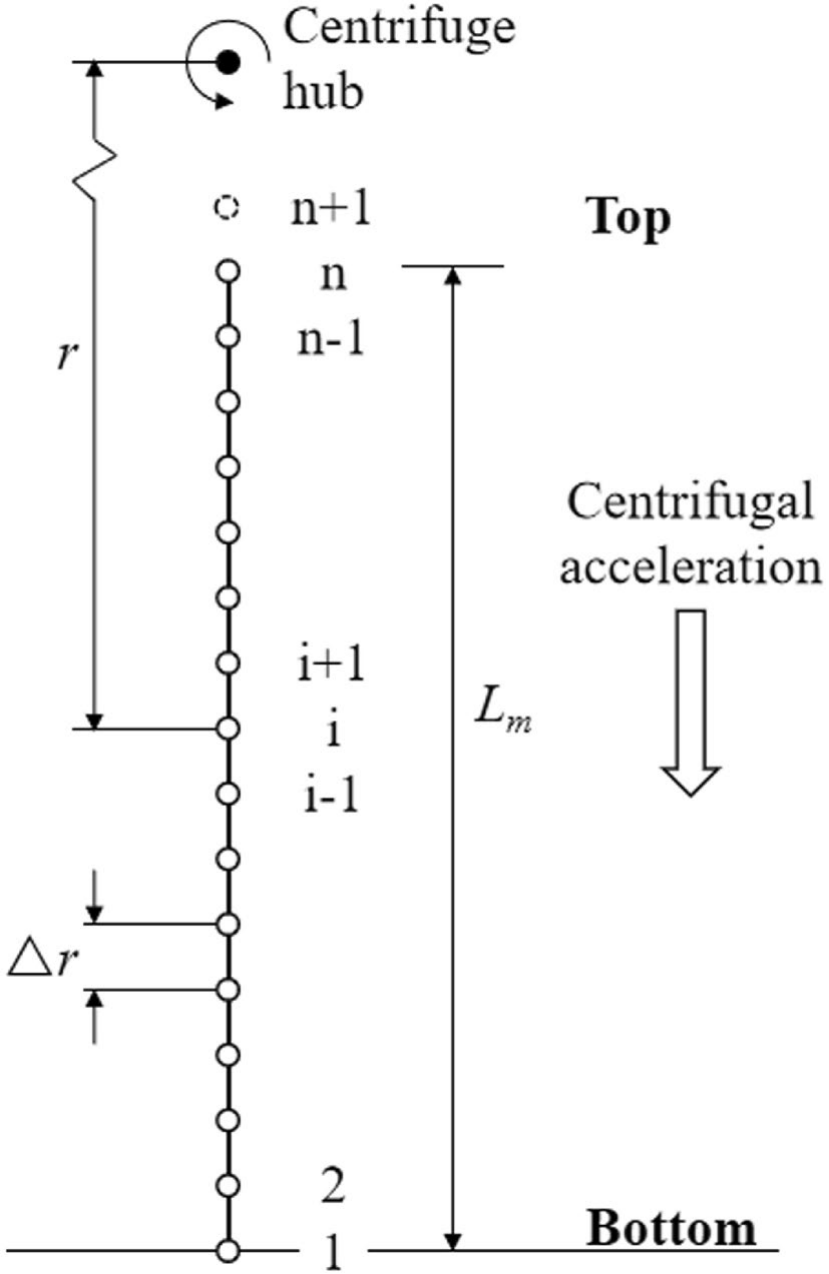
Finite-difference grid blocks for one-dimensional centrifugal model, the (*n* + 1)th node is a virtual one used for boundary condition at the top.

The entire column length of the centrifugal system is divided into *n* finite-difference grids with *n* nodes as shown in Fig. 2 (the (*n* + 1)th node is a virtual one, just used for the flux boundary at the top of the system). For the *i*th node in the system, a fully implicit finite-difference approximation of spatial terms in Eq. (11), using a Picard iteration scheme for linearizing the nonlinear terms, can be described as:

14 $$\begin{aligned} & \frac{1}{\rho g}\frac{\partial }{\partial r}\left[ {K(\psi )_{m} \frac{{\partial \psi_{m} }}{\partial r}} \right] - \frac{{\omega^{2} }}{g}\frac{\partial }{\partial r}\left[ {K(\psi )_{m} r} \right] \\ & \quad \approx \frac{1}{\rho g\Delta r}\left( {\frac{{K_{m,i - 1}^{j + 1,\tau } + K_{m,i}^{j + 1,\tau } }}{2}\frac{{\psi_{m,i - 1}^{j + 1,\tau + 1} - \psi_{m,i}^{j + 1,\tau + 1} }}{\Delta r} - } \right.\left. {{\kern 1pt} \frac{{K_{m,i}^{j + 1,\tau } + K_{m,i + 1}^{j + 1,\tau } }}{2}\frac{{\psi_{m,i}^{j + 1,\tau + 1} - \psi_{m,i + 1}^{j + 1,\tau + 1} }}{\Delta r}} \right) - \frac{{\omega^{2} }}{2g\Delta r}\left( {K_{m,i - 1}^{j + 1,\tau } r_{i - 1} - K_{m,i + 1}^{j + 1,\tau } r_{i + 1} } \right) \\ \end{aligned}$$

where *j* denotes the *j*th discrete time level, Δ*r* is the spatial step [L], *τ* is the Picard iteration level, $K_{m,i}^{j + 1}$ is the hydraulic conductivity of the *i*th node at *j*th time level [L T^−1^], $\psi_{m,i}^{j + 1}$ is the matric suction at that node [M L^−1^ T^−2^], and *K*(*ψ*) is a nonlinear function of *ψ*, which is linearized using a Picard iteration scheme^1^.

A backward Euler approximation, coupled with Picard iteration scheme, is used to discretize the temporal term at the left hand side of Eq. (11):

15 $$\frac{{\partial \theta_{m} }}{{\partial t_{m} }} \approx \frac{{\theta_{m,i}^{j + 1,\tau + 1} - \theta_{m,i}^{j} }}{{\Delta t_{m} }}$$

where Δ*t*_*m*_ is the time step [T]. Using the approach of Celia et al.^29^, $\theta_{m,i}^{j + 1,\tau + 1}$ is expanded using a first-order, truncated Taylor series, in terms of the matric suction arising from Picard iteration about the expansion point ($\theta_{m,i}^{j + 1,\tau }$,$\psi_{m,i}^{j + 1,\tau }$), as:

16 $$\theta_{m,i}^{j + 1,\tau + 1} \approx \theta_{m,i}^{j + 1,\tau } + \left. {\frac{{{\text{d}}\theta }}{{{\text{d}}\psi }}} \right|_{m,i}^{j + 1,\tau } \left( {\psi_{m,i}^{j + 1,\tau + 1} - \psi_{m,i}^{j + 1,\tau } } \right)$$

The specific water capacity of soil is defined as:

17 $$C\left( \psi \right) = \frac{{{\text{d}}\theta }}{{{\text{d}}\psi }}$$

Using Eqs. (15)–(17), the partial time derivative of water content is approximated as:

18 $$\frac{{\partial \theta_{m} }}{{\partial t_{m} }} \approx \left( {\frac{{\theta_{m,i}^{j + 1,\tau } - \theta_{m,i}^{j} }}{{\Delta t_{m} }}} \right) + C_{m,i}^{j + 1,\tau } \left( {\frac{{\psi_{m,i}^{j + 1,\tau + 1} - \psi_{m,i}^{j + 1,\tau } }}{{\Delta t_{m} }}} \right)$$

The first term on the right side of Eq. (18) is an explicit estimate of the partial time derivative of water content, based on the *τ*th Picard level estimates of matric suction. In the second term of the right side of Eq. (18), the numerator of the bracketed fraction is an estimate of the error in the pressure head at node *i* between two successive Picard iterations. Its value diminishes as the Picard iteration process converges. As a result, as the Picard process proceeds, the contribution of the specific water capacity *C*(*ψ*) is diminished^1^.

The finite-difference expressions for the spatial and temporal derivatives of Eqs. (14) and (18) are rearranged by moving all the unknowns on the left side and all the knowns on the right, in agreement with Eq. (11). Using the above implicit finite-difference approximation, the matric suction at the (*j* + 1)th time level and (*τ* + 1)th Picard level is obtained by solving the following linear algebraic equations:

19a $$F_{i}^{3} \psi_{m,i + 1}^{j + 1,\tau + 1} + \left( {F_{i}^{1} + F_{i}^{2} - F_{i}^{3} } \right)\psi_{m,i}^{j + 1,\tau + 1} + \left( { - F_{i}^{2} } \right)\psi_{m,i - 1}^{j + 1,\tau + 1} = F_{i}^{4} + F_{i}^{1} \psi_{m,i}^{j + 1,\tau } - F_{i}^{5}$$

where coefficients $F_{i}^{1}$, $F_{i}^{2}$, $F_{i}^{3}$, $F_{i}^{4}$ and $F_{i}^{5}$ are defined as:

19b $$F_{i}^{1} = \frac{{C_{m,i}^{j + 1,\tau } }}{{\Delta t_{m} }}$$

19c $$F_{i}^{2} = \frac{{K_{m,i - 1}^{j + 1,\tau } + K_{m,i}^{j + 1,\tau } }}{{2\rho g\Delta r^{2} }}$$

19d $$F_{i}^{3} = - \frac{{K_{m,i + 1}^{j + 1,\tau } + K_{m,i}^{j + 1,\tau } }}{{2\rho g\Delta r^{2} }}$$

19e $$F_{i}^{4} = - \frac{{\omega^{2} }}{2g\Delta r}\left( {K_{m,i - 1}^{j + 1,\tau } r_{i - 1} - K_{m,i + 1}^{j + 1,\tau } r_{i + 1} } \right)$$

19f $$F_{i}^{5} = \frac{{\theta_{m,i}^{j + 1,\tau } - \theta_{m,i}^{j} }}{{\Delta t_{m} }}$$

Equation (19) applies to all interior nodes; this equation is modified at boundary nodes to reflect the appropriate boundary conditions as matrix form in below:

20 $${\mathbf{A}}_{m} {{\varvec{\uppsi}}}_{m} = {\mathbf{b}}_{m}$$

where **ψ** is a vector of unknown matric suctions $\psi_{m,i}^{j + 1,\tau + 1}$, **b** is the forcing vector, **A** is a square matrix consisting of the coefficients of the finite-difference Eq. (19). Equation (20) can be solved using a Thomas algorithm.

### Boundary conditions

In most cases, the top boundaries are always set as flux boundaries and the bottom ones are set to be free-drainage faces during centrifugal experiments. According to existing literatures^31^, the free-drainage boundary would be treated as constant pressure head boundary in numerical simulations. Therefore, the boundary conditions used afterwards are as follows: a constant water flux is added at the top and a constant matric suction is fixed at the bottom. Mathematically these conditions can be represented as:

21 $$v_{m} \left( {r = r_{t} ,t_{m} } \right) = q_{b}$$

22 $$\psi_{m} \left( {r = r_{b} ,t_{m} } \right) = \psi_{b}$$

where *q*_*b*_ is the constant flux added at the top [L T^−1^], *ψ*_*b*_ is the constant matric suction at the bottom [M L^−1^ T^−2^], and *r*_*t*_ is the radius of the top [L]. It should be noticed that special attention needs to be paid to flux boundary conditions, since they have an important effect on the stability of the numerical solution, as well as on the overall mass balance^15^. Rewrite Eq. (21) in the form of Eq. (19) during numerical simulations is:

23a $$\left( {F_{n}^{1} + F_{n}^{2} - F_{n}^{3} } \right)\psi_{m,n}^{j + 1,\tau + 1} + \left( {F_{n}^{3} - F_{n}^{2} } \right)\psi_{m,n - 1}^{j + 1,\tau + 1} = F_{n}^{1} \psi_{m,n}^{j + 1,\tau } + F_{n}^{4} - F_{n}^{5} - F_{n}^{3} F_{n}^{6}$$

with

23b $$F_{n}^{6} = 2\Delta r\left( {\frac{{\rho gq_{b} }}{{K_{m,n + 1}^{j + 1,\tau } }} - \rho r_{t} \omega^{2} } \right)$$

### Mass balance calculation

In order to do mass balance analysis for centrifugal modeling of unsaturated flow, water fluxes at the two boundaries and the mass change in the model should be known. The outflow flux at the bottom boundary can be deduced using Taylor series theory, and it’s shown in the form of:

24 $$q_{1}^{j} = K_{m,1}^{j} \left( {\frac{{3\psi_{m,1}^{j} - 4\psi_{m,2}^{j} + \psi_{m,3}^{j} }}{2\rho g\Delta r} - \frac{{r_{b} \omega^{2} }}{g}} \right)$$

where $q_{1}^{j}$ is the outflow flux at *j*th discrete time level [L T^−1^]. While the top is not a flux boundary (e.g., a constant suction boundary with the flux equal to zero), the inflow flux at the top boundary can be calculated as:

25 $$q_{n}^{j} = K_{m,n}^{j} \left( {\frac{{4\psi_{m,n - 1}^{j} - 3\psi_{m,n}^{j} - \psi_{m,n - 2}^{j} }}{2\rho g\Delta r} + \frac{{r_{t} \omega^{2} }}{g}} \right)$$

where $q_{n}^{j}$ is the inflow flux at *j*th discrete time level [L T^−1^]. The mass balance ratio is defined as follows to evaluate the capacity of the simulator^29^:

26 $$MB(e\Delta t_{m} ) = \left| {\frac{{W_{b} }}{{W_{a} }}} \right| = \left| {\frac{{\sum\limits_{j = 1}^{e} {\left( {q_{n}^{j} - q_{1}^{j} } \right)\Delta t_{m} } }}{{\sum\limits_{i = 1}^{n - 1} {\left( {\frac{{\theta_{m,i}^{e} + \theta_{m,i + 1}^{e} }}{2} - \frac{{\theta_{m,i}^{0} + \theta_{m,i + 1}^{0} }}{2}} \right)\Delta r} }}} \right|$$

where $W_{a}$ is the total additional mass in the centrifugal model [L], $W_{b}$ is the total net flux into the model [L], *e* is the total discrete time levels, and $\theta_{m,i}^{0}$ is the initial water content at the *i*th node [L^3^ L^−3^].

### Hydraulic properties of soil

The solution of the Richards’ equation requires knowledge of fluid content and hydraulic conductivity versus matric suction^32^, which are defined by the soil moisture characteristic curve and the permeability function, respectively. In this study, the van Genuchten model^33^ is used to describe the soil moisture characteristic curve as:

27 $$\Theta = \frac{{\theta - \theta_{r} }}{{\theta_{s} - \theta_{r} }} = \left[ {\frac{1}{{1 + \left( {\alpha h} \right)^{{n_{v} }} }}} \right]^{{m_{v} }}$$

where Θ is effective saturation (dimensionless), *α* [L^−1^], *n*_*v*_ (dimensionless) and *m*_*v*_ = 1 − (1/*n*_*v*_) (dimensionless) are van Genuchten model parameters, *θ*_*s*_ and *θ*_*r*_ are saturated and residual water content [L^3^ L^−3^], respectively, *h* is water pressure head [L] and can be calculated with *h* = *ψ*/(*ρg*). The permeability function has many forms, Gardner^34^ and Mualem^35^ models are the most popular ones, and their mathematical expressions are as follows:

28 $$K(\psi ) = \frac{{K_{s} }}{{\left( {\psi /\psi_{o} } \right)^{\beta } + 1}}$$

29 $$K\left( \Theta \right) = K_{s} \left\{ {1 - \left[ {1 - \Theta^{{\left( {1/m_{v} } \right)}} } \right]^{{m_{v} }} } \right\}^{2} \Theta^{l}$$

where *K*_*s*_ is saturated hydraulic conductivity of the porous media [L T^−1^], *ψ*_*o*_ [M L^−1^ T^−2^] and *β* (dimensionless) are Gardner model parameters, and *l* is a pore connectivity parameter (dimensionless). The pore connectivity parameter *l* in the hydraulic conductivity function was estimated by Mualem^35^ to be 0.5 as an average for many types of soils. Both Gardner’s and Mualem’s models are used in this manuscript, while only Mualem’s model (i.e. Eq. 29) is used to check the feasibility of doing unsaturated centrifugal modeling.

## Published physical models for benchmarking

**Figure 3:**
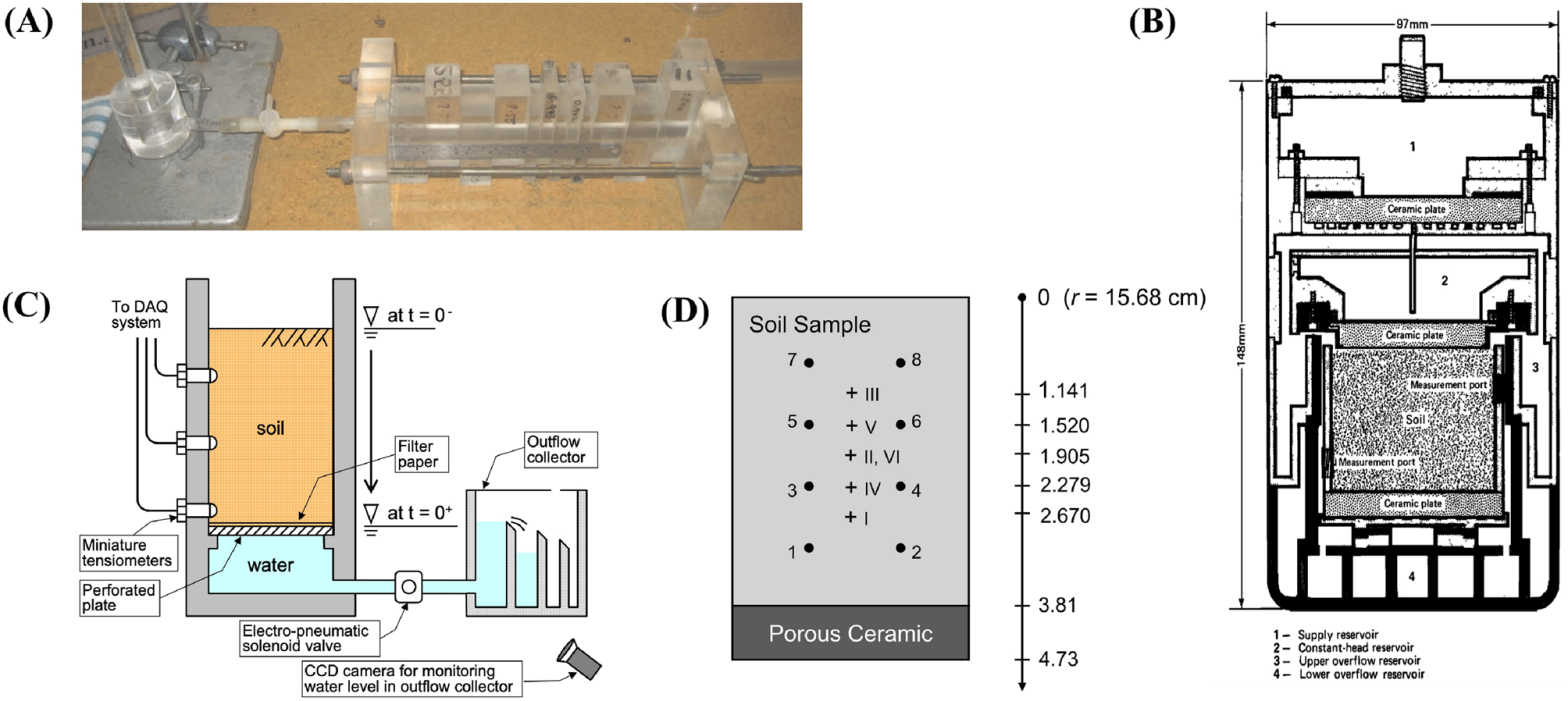
Diagrams of the centrifugal apparatuses used in the four cases: (**A**) Case 1, soil column attached to the Marriotte bottle (adopted from Kirkham^36^), (**B**) Case 2, cross-section view of the experimental apparatus (adopted from Nimmo et al.^22^), (**C**) Case 3, cross-section view of the centrifugal experiment setup (adopted from Nakajima and Stadler^14^), and (**D**) Case 4, sketch of the soil sample, location of electrodes (Arabic numerals) and assumed locations for water content measurements (Roman numerals) (adopted from Šimůnek and Nimmo^15^). Water contents for locations I to VI were calculated by analyzing signal from different sets of four electrodes, namely, (1, 2, 3, 4), (3, 4, 5, 6), (5, 6, 7, 8), (1, 2, 5, 6), (3, 4, 7, 8) and (1, 2, 7, 8), respectively.

The performance of the numerical model presented above is compared with four published experimental datasets, each representing a different scenario. The experimental setups of the four cases are shown in Fig. 3, and the parameters used in the numerical simulations are listed in Table 1.

**Table 1:** Summary of simulation parameters for Cases 1–4.

Case	Type of experiment	*L*_*m*_/cm	*R*_*b*_/cm	*K*_*s*_/m s^−1^	van Genuchten model parameters				Permeability function	*N*	△*r*/mm	△*t*/s	Source
					*θ*_*r*_	*θ*_*s*_	*α*/m^−1^	*n*_*v*_					
1	Absence of elevation potential	25.0	–	2.117 × 10^–5^	0.100	0.565	1.80	1.408	Mualem model (*l* = 2.956)	–	1	1	Kirkham^36^
2	Steady-state profiles	4.7^a^	22.1^a^	2.92 × 10^–5^	0.0672	0.38	4.105	2.463	Gadner model (*ψ*_*o*_ = − 1.499 Kpa and *β* = 4.5398)	194	0.94	36	Nimmo et al.^22^ and Nimmo^7^
3	Transient unsaturated flow processes	25.4	182	4.82 × 10^–5b^	0.1045^b^	0.37	1.245^b^	8.725^b^	Mualem model (*l* = 0.5)	10	5.08	3.6	Nakajima and Stadler^14^
		12.7		3.66 × 10^–5b^	0.1045^b^	0.37	1.285^b^	8.740^b^		20	2.54	1.8	
		6.35		2.23 × 10^–5b^	0.0535^b^	0.37	1.530^b^	8.550^b^		40	1.27	0.36	
4	Transient unsaturated flow with multi-rotation	3.81	19.49	7.30 × 10^–7^	0.043	0.220	1.76	2.38	Mualem model (*l* = 0.5)	See Table 2	0.762	10	Šimůnek and Nimmo^15^ and Nimmo et al.^22^

^a^Not directly give in the paper, but get from the figure of it.

^b^Mean values of the two parallel experiments listed in Nakajima and Stadler^14^.

**Table 2:** Sequences of centrifuge speeds of Run 1 in Case 4^15^.

*t*/s	10,249	64,129	72,353	80,352	92,299	144,744	169,222
*ω*/rad s^−1^	46.1	57.6	70.2	74.3	104.7	136.1	230.4
*N*	36.8	57.4	85.2	95.5	189.6	320.4	918.1

### Case 1: suction forced unsaturated flow when ω = 0

Data used in Case 1 come from Kirkham^36^ (Fig. 3A), where the unsaturated flow is forced by suction gradient with the absence of elevation potential (*ω* equals to zero). In this case, the centrifugal system can be considered as a horizontal column experiment. The horizontal column was constructed of acrylic sections which vary from 0.9 to 2.6 cm in length. The acrylic material enables the advance of the wetting front to be observed and the section orientation allows rapid partitioning of the column at the end of the experiment by pushing down on column sections. The total length of the column is 25 cm filled with the Ferrosol soil. One end of the column was attached to a Marriotte bottle, and the other end was kept open. As it was destructive sampling, six experiments were conducted to get a time series data of water flow. The initial and boundary conditions were almost the same with slight differences, and average values are used here for simulations. The initial water content of soil column was uniformly set to be 0.155 cm^3^/cm^3^, the end which was attached to the Marriotte bottle is treated as a constant suction boundary with the value of 0 Kpa, and the other end is treated as a free-drainage boundary.

### Case 2: steady-state profiles

Data used in Case 2 come from Nimmo et al.^22^ (Fig. 3B), and it is selected to verify the prediction of the steady-state profiles. In this case, an internal flow control (IFC) apparatus to measure unsaturated hydraulic conductivity in a relatively short time was developed and Darcy’s law under low hydraulic conductivity was tested. A cylindrical sample with the diameter of 50 mm and height of 47 mm was filled with Oakley sand, and then was saturated and put on the apparatus. The distance between the bottom of the sample and the centrifuge axis was 221 mm, and an angular speed, 100 rad/s (*N* ≈ 194), was applied to the sample. During centrifugation, water was continuously supplied at the top and a constant matric suction (− 10 Kpa) was established at the bottom. The water used to saturate the soil and applied to the sample was a deaired solution of 0.01 N CaSO_4_ and 0.01 N CaSeO_4_, which was designed to inhibit microbiological growth and to prevent changes in soil structure due to dispersion of clay. The sample was sliced into 1.25-mm-thick pieces to obtain the moisture content profile when the steady-state was achieved. The van Genuchten model parameters of Oakley sand were obtained from data fitting in Nimmo^7^.

### Case 3: transient unsaturated flow

Data used in Case 3 come from Nakajima and Stadler^14^ (Fig. 3C), and it is selected to verify the prediction of transient unsaturated flow processes. The original physical model was intended to estimate unsaturated soil parameters by using one-step outflow tests with the help of a 2-m radius geotechnical centrifuge which is set at the Idaho National Laboratory. An apparatus which allows suction pressure heads within the samples and cumulative outflow to be measured was used. The apparatus was filled with dry fine Ottawa sand and saturated by deaired water in a large vacuum chamber for all the experiments. Then, the container filled with soil was placed on the centrifuge to desired accelerations. The top of the samples were set as zero-flux boundaries, and the bottom were kept contacting with water table while the experiments were going. Cumulative outflow data and suction pressure heads during the processes were collected, and then the unsaturated soil parameters were estimated based on the collected data. These experiments were conducted under three different *g*-levels (10 *g*, 20 *g*, and 40 *g*), but the same prototype was modeled.

### Case 4: unsaturated flow in multi-rotation experiments

Data in Case 4 come from Šimůnek and Nimmo^15^ (Fig. 3D), and it is chosen to verify the prediction of transient unsaturated flow while doing multi-rotation experiments with a centrifuge. The data were collected in three multi-rotation experiments, which were marked as Run 1, Run 2 and Run 3, respectively. The apparatus used to do the experiments was the IFC apparatus which developed by Nimmo et al.^22^, and eight electrodes were buried for water content measurements at five different depths with six data channels. Oakley sand was packed in the apparatus and was saturated. Afterwards, experiments were carried out and data were collected meanwhile with zero-flux top-boundary and constant suction bottom-boundaries. Equilibrium and transient analyses were conducted and then unsaturated hydraulic parameters were estimated.

## Results

**Figure 4:**
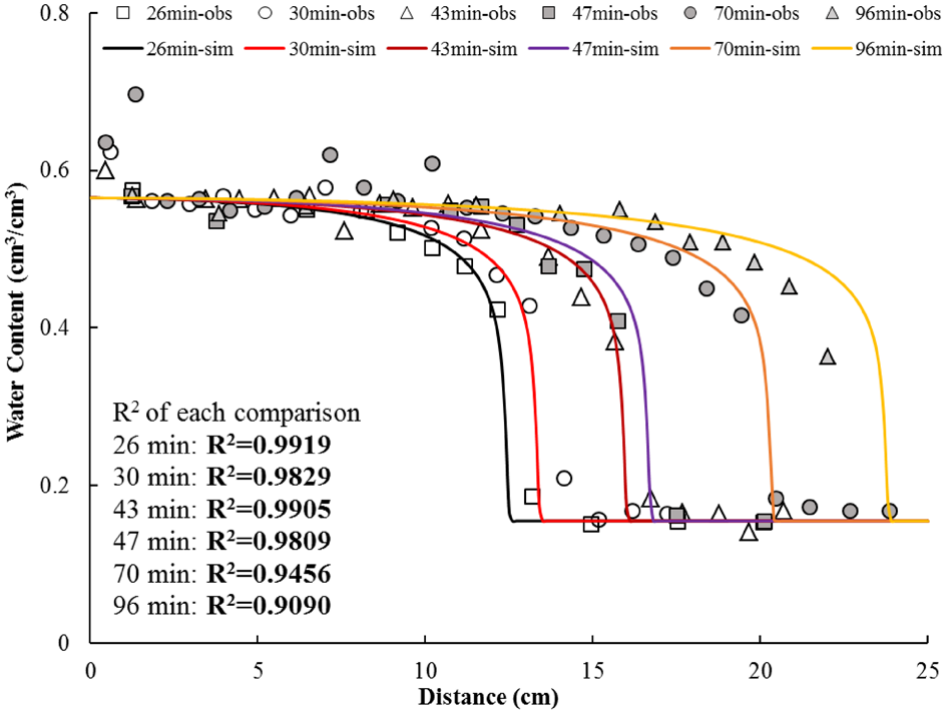
Comparison between data from Kirkham^36^ (points with the suffix “-obs”) and simulated results by this study (solid lines with the suffix “-sim”) for suction dominant unsaturated flow in horizontal column experiments. The coefficients of determination (R^2^) for all the comparisons are also listed in the figure.

In Case 1, the comparison between Kirkham’s data^36^ of unsaturated flow dominated by matrix suction gradient and the simulation results are shown in Fig. 4. The unsaturated flow processes are simulated by the numerical model with parameters and adjusting conditions listed in Table 1. A good agreement (R^2^ values of all six comparisons are larger than 0.9) is observed, which illustrates that the numerical model could be applied for this situation.

**Figure 5:**
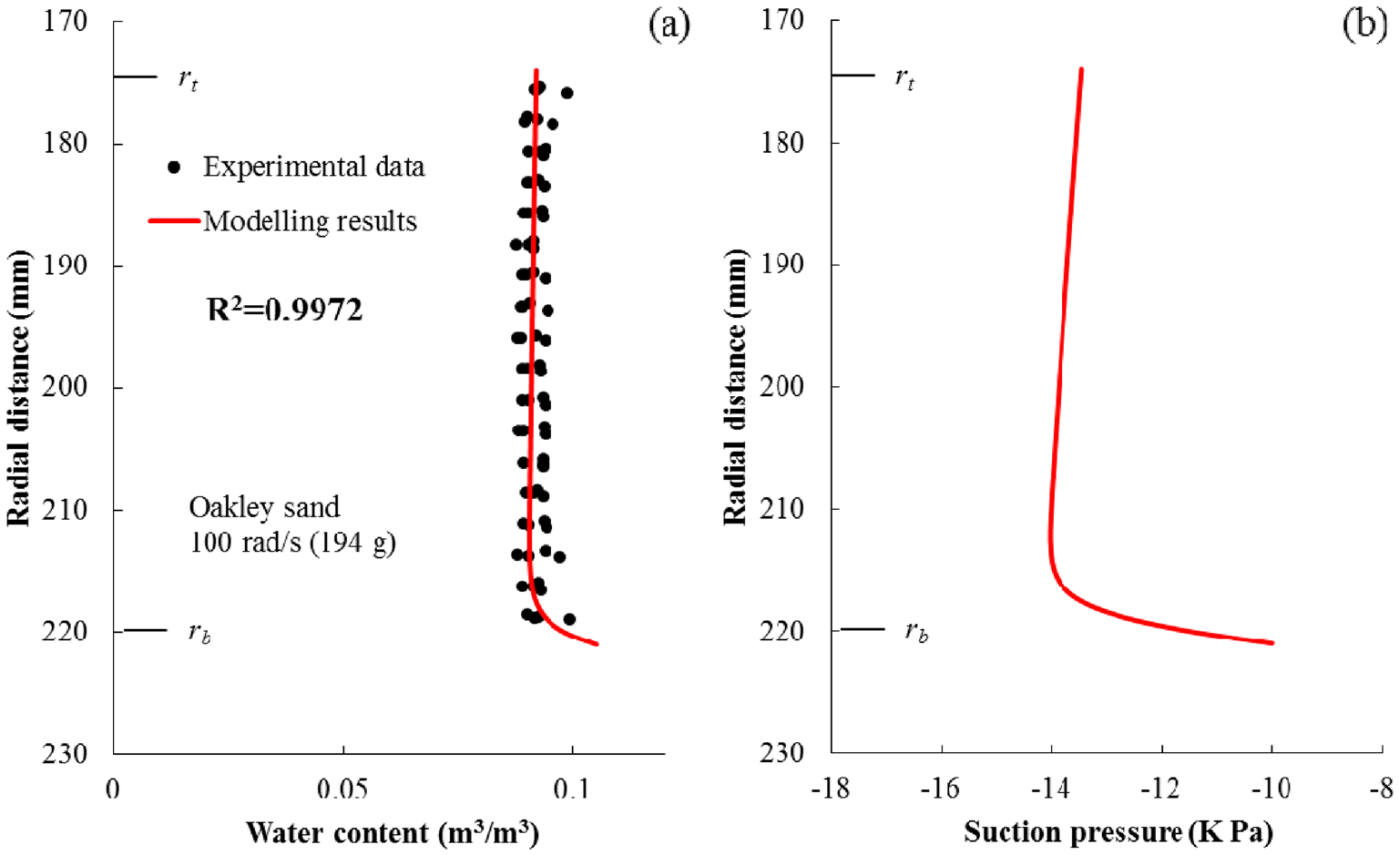
Simulation of soil moisture content profiles under steady-state condition: (**a**) moisture content profile predicted by the numerical model and comparison with data from Nimmo et al.^22^, (**b**) predicted suction profile. The coefficient of determination (R^2^) is also listed in the figure.

In Case 2, results predicted by the numerical model and data collected by Nimmo et al.^22^ are compared in Fig. 5a. It is illustrated that there is a good agreement between the model simulation and the observed data in terms of moisture content profile. The R^2^ value between the simulated value and observed data is 0.9972. Furthermore, the steady-state suction profile was also predicted and was shown in Fig. 5b.

**Figure 6:**
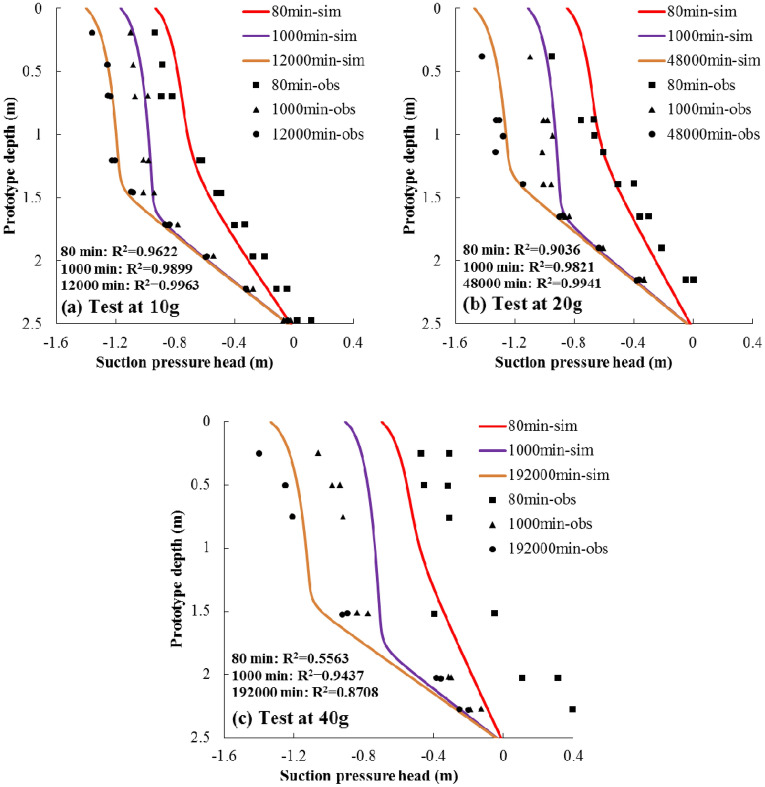
Simulation results (solid lines suffixed with “-sim”) versus transient flow data (points suffixed with “-obs”) collected by Nakajima and Stadler^14^, the prototype time shown in the figures has a relationship with centrifugal modeling time and the mathematical expression is: *t*_*p*_ = *N*^2^*t*_*m*_. The coefficients of determination (R^2^) for all the comparisons are also listed in the figure.

In Case 3, the predicted transient flow is compared with experimental results presented by Nakajima and Stadler^14^ in Fig. 6. It is illustrated that the agreement between simulated and observed results are acceptable. The R^2^ values of the most comparisons are larger than 0.9, while the R^2^ values of the comparisons of 80 min and 192,000 min at *g*-level of 40 *g* are 0.5563 and 0.8708, respectively. Factors resulting in poor match of testing at 40 *g* are caused by non-ideal controlled boundaries and the limitation of tensiometers’ performance. According to Fig. 6, it is difficult for water to be discharged from the bottom of samples at the starting phase of the experiment. The positive values of suction pressure heads near the bottom at 80 min (prototype time) indicate that water is ponding there. Besides, rapid pore water pressure drop would occur when testing under higher *g*-level, eventually inducing cavitation of the ceramic attached on the tensiometers^14^, which causes poor performance of these inset tensiometers.

**Figure 7:**
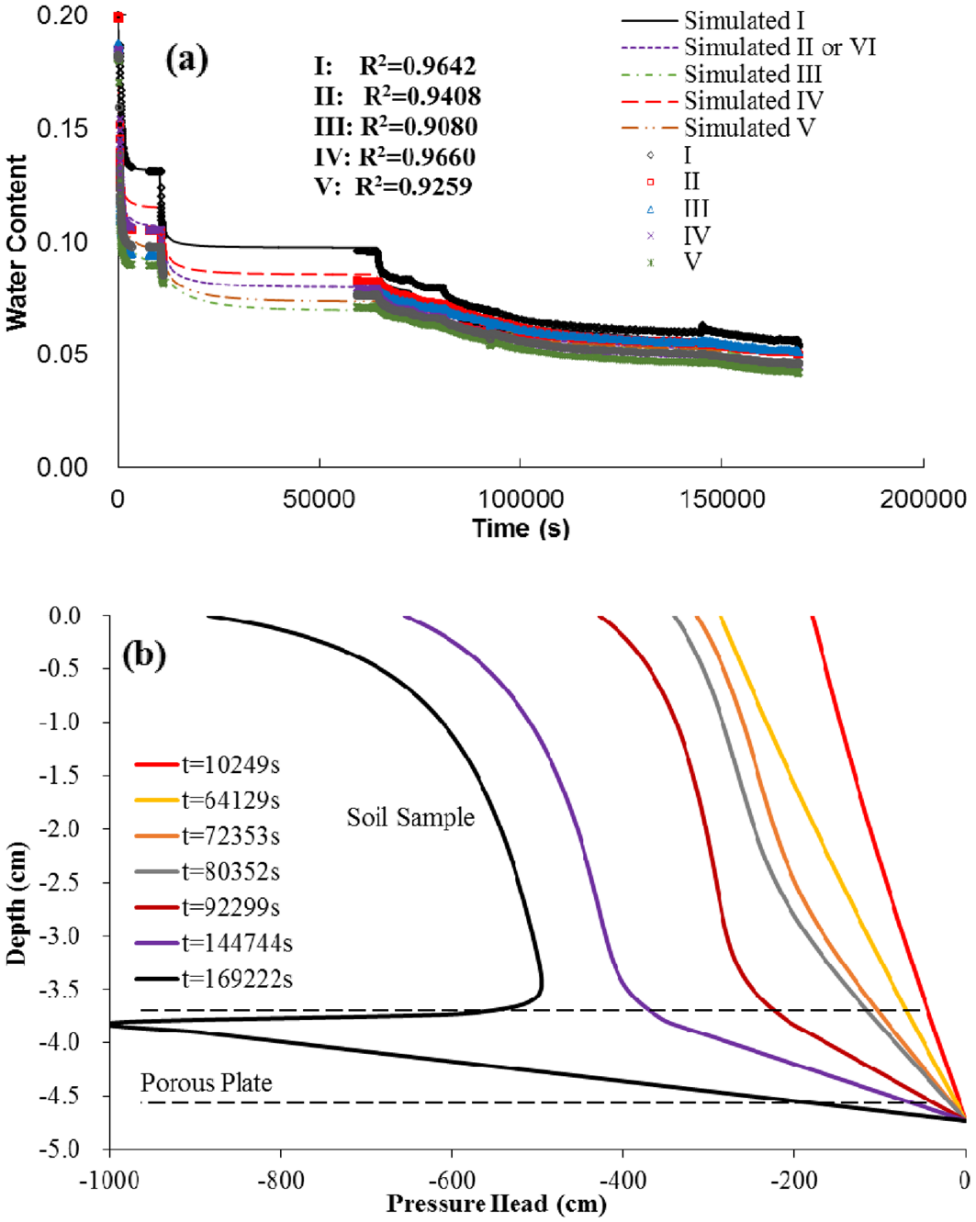
(**a**) Predicted water content changing at five different depths by the numerical model versus data (points) observed by Šimůnek and Nimmo^15^ for Run 1, where I–VI represent the assumed locations for water content measurements in Šimůnek and Nimmo^15^. The coefficients of determination (R^2^) for all the comparisons are also listed in the figure, and (**b**) simulated pressure heads profiles at times when the rotation speed was changed.

Three separate runs from re-saturation with different rotation speeds were considered and simulated in Case 4. As these runs have similar intentions, only Run 1 of Case 4 is selected to test the unsaturated flow in multi-rotation experiments in this study, and the sequences of centrifuge speeds are given in Table 2. It should be noted that the value of constant suction at soil bottom varies at times when the rotation speed is changed. Since a 9.2 mm porous plate was set at soil bottom for supporting during the experiments, only the plate’s bottom was kept contact with the water table. 9.2 mm is sufficiently long for the whole plate to be saturated within a centrifugal field, so the suction at the soil bottom (contact with the top face of the plate) will not be zero. In order to deal with it, an assumption was made for the simulation, that is, every point on the plate has a same fluid potential. In that case and based on Eq. (6), the constant suction at the soil bottom can be obtained:

30 $$\psi_{b} = - \frac{1}{2}\rho \omega^{2} H\left( {2r_{b} + H} \right)$$

where *H* is the thickness of the plate [L]. With the bottom boundary condition solved, unsaturated flow in Run 1 is predicted then, and the simulation results are compared with the data from Šimůnek and Nimmo^15^ in Fig. 7. The R^2^ values of these five depths between the simulated values and observed data are 0.9642, 0.9408, 0.9080, 0.9660 and 0.9259, respectively, which are all larger than 0.9. This indicates that a good agreement of the water content changing at five depths can be observed and the predicted pressure head profiles at times when the rotation speed is changed are exactly the same as data presented by Šimůnek and Nimmo^15^.

## Discussion

### Validation of the numerical model by four physical models

**Figure 8:**
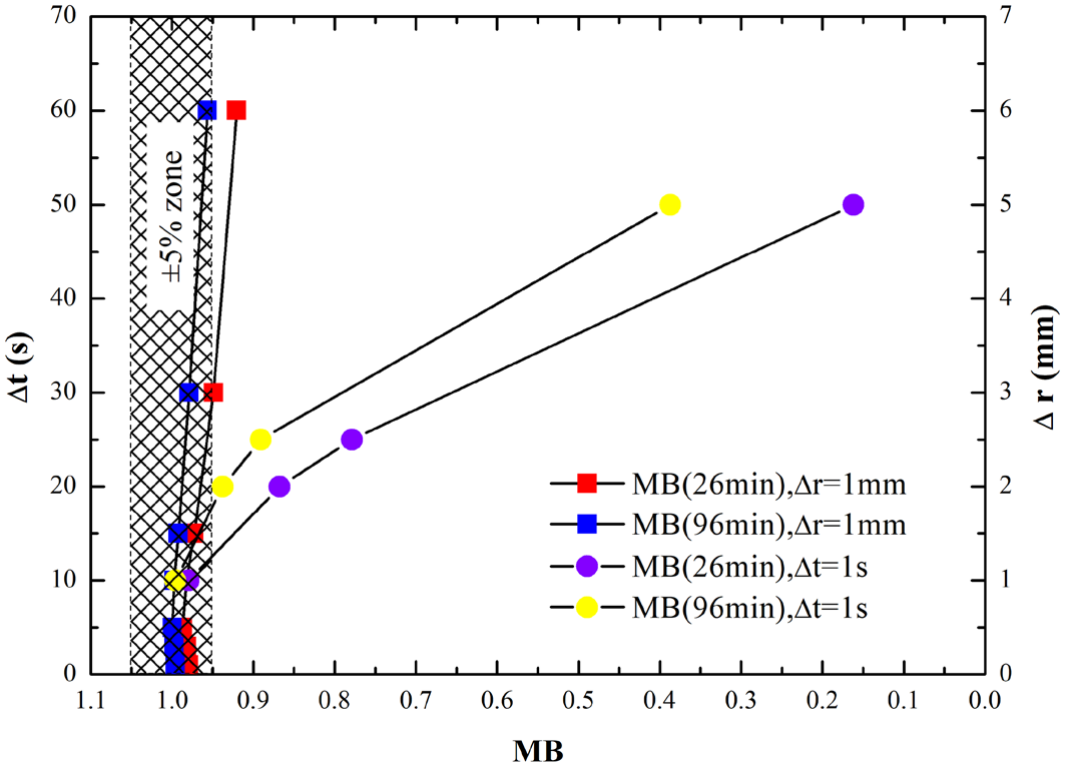
Mass balance performance of the numerical model in Case 1. Results indicated with rectangles are calculated with △*r* = 1 mm and △*t* varied from 1 to 60 s, and results indicated by circles are calculated with △*t* = 1 s and △*r* varied from 1 to 5 mm.

The numerical model developed in the present study was validated by four benchmark cases, and it was shown that the numerical model can be well applied in simulating unsaturated flow of centrifugal systems over a broad range of problems. However, the numerical model also showed certain limitations when predicting unsaturated flow with sufficient accuracy. Taking Case 1 as an example, a further inspection was carried out on the simulated mass balance in connection with the model discretization (Eq. 26 applied), and the results are shown in Fig. 8. According to Fig. 8, the numerical model showed good mass conservation when both time and space intervals are small. The performance of the numerical model is more sensitive to the spatial discretization than to the temporal discretization. When the time step varied from 1 to 60 s, the numerical solution exhibited mass balance error less than 5% at 96 min. On the contrary, the mass balance errors are more than 10% and 60% at 96 min when the space spans are 2.5 mm and 5 mm, respectively. Therefore, it is suggested that a fine spatial discretization is necessary to ensure the quality of simulation. It is noted that case 1 had no inertial acceleration added to the column, and since angular speed is not zero, a significant finer spatial discretization is needed to accommodate large gradients at early times of the simulation to achieve smaller mass balance errors, especially at the outflow boundary^15^.

It is noted from Fig. 5b that when the steady-state condition is achieved, the distributions of moisture content and suction towards the sample top are almost uniform. This phenomenon was also observed by other studies^16,26,31^, and the larger the *N* value of gravity level is, the more obvious the phenomenon is^31^. In that case, the suction gradient in Eq. (8) can be ignored under steady-state conditions

31 $$v_{m} = - q_{b} = - K(\psi )_{m} \frac{{r\omega^{2} }}{g}$$

Express *K* as a function of moisture content, *θ*, and rearrange Eq. (32):

32 $$K(\theta )_{m} = \frac{{q_{b} g}}{{r\omega^{2} }}$$

Equation (32) is the basic equation of Nakajima and Stadler^14^ to measure unsaturated hydraulic conductivity. According to Eq. (32), the unsaturated hydraulic conductivity can be calculated with a known water flux supplied and angular speed, and the corresponding water content could be detected after the experiment. Furthermore, a different data set (*K*, *θ*) can be obtained while changing the rotation speed with other conditions kept the same, so it can be used to get soil moisture characteristic curves conveniently^31^.

### Validity of using a numerical model for 1D centrifugal modelling

Several previous studies have investigated the uncertainty of using a numerical model for 1D centrifugal modeling. Goforth et al.^19^ deduced the formula of Darcy’s law, which is similar to Eq. (8), to describe the centrifugal fluid mechanism by doing a force equilibrium analysis on a fluid volume. They pointed out that the seepage velocity is directly proportional to the *g*-level only if the pressure gradient (i.e., ∂*ψ*_*m*_/∂*r*) is zero, so scale factor for the seepage velocity maybe wrong. Furthermore, fluid flux in soil is dominated by suction gradients, which can be 10–1000 times greater than the gradient due to gravity^37^. In that case, Goforth et al.^19^ commented that there is no advantage of modeling unsaturated flow in a centrifugal field and it may not be feasible, but no useful data were collected by them to support such claim. Poulose et al.^20^ investigated moisture migration in silty soil by using a small centrifuge. They showed that the models may be valid only for saturated soil by comparing data collected at three different *g*-levels, so they agreed with Goforth et al.^19^’s comments. However, several applications of centrifugal modeling under unsaturated situations are shown in literatures^5–8^ and most of them give tacit consent to that it is feasible to do it. Therefore, it is time to make the uncertainty more clear, and the algorithm presented above is used for this purpose.

**Figure 9:**
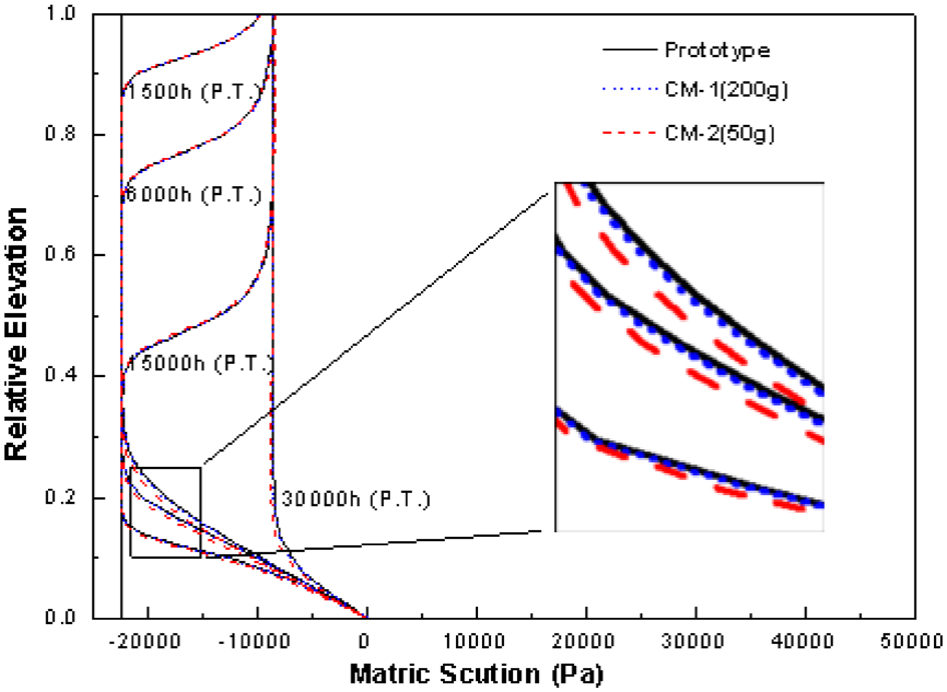
Numerical verification of the feasibility for centrifuges to be applied to model unsaturated flow using Hydrous 1-D software package, where prototype refers to the prototype model and CM represents centrifugal model.

Through different methods (i.e., dimensionless analysis, inspectional analysis, and analytical solutions’ comparison), the scale factors for unsaturated flow are *N*, 1/*N*, and 1/*N*^2^, which are corresponding to the seepage velocity, dimensions, and time between the centrifugal system and the prototype, respectively. In order to verify whether centrifugal modeling could reproduce unsaturated flow of the prototype, these scale factors are assumed to be right at first. Afterwards, an infiltration scene is simulated by the numerical model presented above, and Eq. (12) is also numerically solved to predict the unsaturated flow under the same situation (i.e. prototype). The numerical scheme of Eq. (12) can refer to the above-mentioned algorithm, which is based on the Clement et al.^1^ approach. In order to judge whether the centrifugal experiment simulation can correctly reproduce the prototype, Hydrus 1-D software package is used to directly predict the unsaturated water transfer process in the full-scale prototype. The simulated scene is water infiltrating through a uniform, unsaturated, low permeable soil column, which has a saturated hydraulic conductivity of 1 × 10^–7^ m/s. The initial water content throughout the column is 0.22, the column top is a flux boundary and the bottom is kept wet during the migration processes. The input and modeling parameters of the centrifugal system under different *g*-levels and of the prototype are listed in Table 3, and these scale factors are already reflected in the values. A comparison of simulated results of centrifugal systems and the prototype is made in Fig. 9. It can be seen in Fig. 9 that the unsaturated flow in the scaled-down centrifugal systems are almost the same as the prototype except for the lower part of the column, which preliminary illustrates that these scaling parameters are useful factors and centrifugal modeling can be used in studying of unsaturated flow processes.

**Table 3:** Parameters used in the centrifugal model and the prototype simulated by Hydrous 1-D software package.

Parameters	*N*	*L*/m	*r*_*b*_/m	*θ*_*s*_	*θ*_*r*_	*n*_*v*_	*α*/m^−1^	*l*	*K*_*s*_/m s^−1^	Top flux/m s^−1^	△*r* or △*z*/mm	△*t*/s	Observed time nodes/h
CM-1^a^	200	0.05	1.5	0.4	0.1	2	1	0.5	1 × 10^–7^	2 × 10^–6^	1	0.18	0.0375, 0.15, 0.375, and 0.75
CM-2^a^	50	0.2	1.5	0.4	0.1	2	1	0.5	1 × 10^–7^	5 × 10^–7^	4	2.88	0.6, 2.4, 6, and 12
Prototype	–	10	–	0.4	0.1	2	1	0.5	1 × 10^–7^	1 × 10^–8^	200	7200	1500, 6000, 15,000, and 30,000

^a^CM represents centrifugal model.

### Use of centrifugal modeling under unsaturated flow condition

As mentioned above, Goforth et al.^19^ pointed out that seepage velocity is directly proportional to the *g*-level only if the pressure gradient is zero, but the simulation results of the hypothetical scene show that it may be incorrect. This is because there could be another situation where the pressure gradient is also proportional to the *g*-level itself, which guarantees that the seepage velocity is proportional to the *g*-level. According to Fig. 9, the matric suction at the same corresponding place is the same for both centrifugal systems and the prototype, and it should be noticed that the dimension is deduced by a factor of 1/*N*, so the pressure gradient between any two specified points in the centrifugal system would be *N* times larger than that in the prototype. That means the pressure gradient is proportional to the *g*-levels, and the scale factor of the seepage velocity is correct. Goforth et al.^19^ commented that it may not be feasible to model unsaturated flow with a centrifuge. One possible reason could be that they considered that centrifugal field would enhance the status of elevation potential gradient. The proportions of elevation potential gradient in the total driving force for unsaturated flow in the centrifugal system and the prototype are:

33 $$\eta_{m} = \frac{{\rho r\omega^{2} }}{{\rho r\omega^{2} - \frac{{\partial \psi_{m} }}{\partial r}}} = \frac{\rho Ng}{{\rho Ng - \frac{{\partial \psi_{m} }}{\partial r}}}$$

34 $$\eta_{p} = \frac{\rho g}{{\rho g - \frac{{\partial \psi_{p} }}{\partial z}}}$$

It is known that the pressure gradient is proportional to the *g*-level, so *η*_*m*_ equals to *η*_*p*_, which means that centrifugal field will not change the status of elevation potential gradient. By using Poiseuille’s equation of capillary flow, Lord^38^ checked the capillary flow under different situations in the geotechnical centrifuge, and the results show that the scale laws for time and dimension of one or two-dimensional unsaturated flow maybe correct. Therefore, Goforth et al.^19^’s comments may be incorrect. In addition, it should also be highlighted that the numerical verification result shown in Fig. 9 proves that scale factors for time, seepage velocity, and dimension are correct without assuming that the centrifuge acceleration is uniformly distributed, and this method is not limited to the steady-state conditions like the work of Dell'avanzi et al.^16^.

Furthermore, the slight differences between the lower part of centrifugal system and prototype could be noticed in Fig. 9. The unsaturated flow at the lower part of the centrifugal system lagged behind the prototype as the matric suctions at this part are smaller than the prototype. This happened because the method used to handle spatial distance with the same *N* value for the whole centrifugal system. According to Eqs. (3) and (13), the *g*-level value at the lower part of the centrifugal system should be larger than *N*, and this would cause the size of the lower part to be reduced compared with the real space distance. For the same reason, the size of the upper part is caused to be enlarged compared with the real space. The error due to this (as shown in the inset of Fig. 8) is still within an acceptable small range. Two methods could be applied to reduce the error caused by the uneven distribution of acceleration, conducting centrifugal experiments with large *N* values or treating the space distance with the exact g-level values for the different points on the centrifugal system.

It should be noted that the parameters used in the numerical model (namely, the parameters presented in Tables 1, 2, 3) were assumed constant as the *g*-level changed. Based on this, it can be concluded that theoretically centrifugal modeling is feasible to be applied for modeling unsaturated flow. However, there could be unmatched results between model simulations and real data collected from physical experiments^20^. In real physical systems, various factors could bring in large experimental uncertainty for unsaturated flow, such as poorly controlled boundaries, the emergence of compaction, performance limitations of the installed sensors, variations in the preparation of soil samples, etc. It can be seen from Case 3, the improperly controlled bottom boundary caused strange data at 80 min (prototype time), and the limitation of tensiometers resulted in almost failing experiments at 40 g. Samples with strong plasticity (such as clay) trend to be compacted by the centrifugal acceleration, and result in a change of soil water characteristics, therefore the experimental data can vary a lot. As the modeling method is used by Poulos et al.^20^, several experiments with different *g*-levels need to be completed for a special prototype, and the differences between soil preparations could be a reason for variations in the data reported in their study.

## Conclusions

Centrifugal modeling has been used for investigating the flow and solute transport behavior in both saturated and unsaturated soil in the past. However, it has been questioned whether this approach is suitable for applying under unsaturated conditions. In the present study, a numerical model for one-dimensional unsaturated flow in centrifugal systems was developed and verified using four published benchmark datasets.

The newly developed numerical model was able to recreate the four benchmark datasets with reasonable accuracy. Therefore, it is suggested that the numerical model can be used to predict unsaturated flow processes in centrifugal systems when certain criteria are met.

The numerical model was able to close the water budget when spatial and temporal intervals are sufficiently small. The model is more sensitive to spatial discretization than to temporal discretization. Therefore, finer spatial discretization is advised (e.g. 1 mm) to ensure high quality simulation.

It is feasible to study flow processes occurring within the unsaturated zone using centrifugal experiment. The concerns raised by Goforth et al.^19^ may not have many impacts since the matric suction gradient is directly proportional to the gravity level *N*, and the centrifugation will not strengthen the importance of the position potential energy gradient in the process of driving the unsaturated flow. The similarity ratios of seepage velocity (*N*), dimensions (1/*N*) and temporal size (1/*N*^2^) proposed by the predecessors are indeed reasonable, and these ratios are used effectively in the examples discussed in this study.

The uneven distribution of acceleration in the simulation of centrifugal experiment could cause water arrival at the bottom of the soil column lag behind the prototype. When using the same centrifugal equipment to simulate the same prototype, the use of higher centrifugal acceleration can reduce this type of lag.

## Data Availability

The datasets used and/or analyzed during the current study available from the corresponding author on reasonable request.
